# Azithromycin Resistance through Interspecific Acquisition of an Epistasis-Dependent Efflux Pump Component and Transcriptional Regulator in Neisseria gonorrhoeae

**DOI:** 10.1128/mBio.01419-18

**Published:** 2018-08-07

**Authors:** Crista B. Wadsworth, Brian J. Arnold, Mohamad R. Abdul Sater, Yonatan H. Grad

**Affiliations:** a Department of Immunology and Infectious Diseases, Harvard T. H. Chan School of Public Health, Boston, Massachusetts, USA; b Department of Epidemiology, Harvard T. H. Chan School of Public Health, Boston, Massachusetts, USA; c Division of Infectious Diseases, Brigham and Women’s Hospital, Harvard Medical School, Boston, Massachusetts, USA; Emory University School of Medicine; Harvard Medical School

**Keywords:** *Neisseria gonorrhoeae*, antibiotic resistance, efflux pump, epistasis, gonorrhea, macrolide

## Abstract

Mosaic interspecifically acquired alleles of the multiple transferable resistance (*mtr*) efflux pump operon correlate with increased resistance to azithromycin in Neisseria gonorrhoeae in epidemiological studies. However, whether and how these alleles cause resistance is unclear. Here, we use population genomics, transformations, and transcriptional analyses to dissect the relationship between variant *mtr* alleles and azithromycin resistance. We find that the locus encompassing the *mtrR* transcriptional repressor and the *mtrCDE* pump is a hot spot of interspecific recombination introducing alleles from Neisseria meningitidis and Neisseria lactamica into N. gonorrhoeae, with multiple rare haplotypes in linkage disequilibrium at *mtrD* and the *mtr* promoter region. Transformations demonstrate that resistance to azithromycin, as well as to other antimicrobial compounds such as polymyxin B and crystal violet, is mediated through epistasis between these two loci and that the full-length mosaic *mtrD* allele is required. Gene expression profiling reveals the mechanism of resistance in mosaics couples novel *mtrD* alleles with promoter mutations that increase expression of the pump. Overall, our results demonstrate that epistatic interactions at *mtr* gained from multiple neisserial species has contributed to increased gonococcal resistance to diverse antimicrobial agents.

## IMPORTANCE

Neisseria gonorrhoeae is the sexually transmitted bacterial pathogen responsible for more than 100 million cases of gonorrhea worldwide each year. The incidence of resistance to the macrolide azithromycin has increased in the past decade; however, a large proportion of the genetic basis of resistance remains unexplained. This study is the first to conclusively demonstrate the acquisition of macrolide resistance through *mtr* alleles from other *Neisseria* species, demonstrating that commensal *Neisseria* bacteria are a reservoir for antibiotic resistance to macrolides, extending the role of interspecies mosaicism in resistance beyond what has been previously described for cephalosporins. Ultimately, our results emphasize that future fine-mapping of genome-wide interspecies mosaicism may be valuable in understanding the pathways to antimicrobial resistance. Our results also have implications for diagnostics and public health surveillance and control, as they can be used to inform the development of sequence-based tools to monitor and control the spread of antibiotic-resistant gonorrhea.

## INTRODUCTION

The causal agent of gonorrhea, Neisseria gonorrhoeae, is a Gram-negative diplococcus and an exclusively human pathogen. The prevalence of N. gonorrhoeae with resistance to azithromycin has dramatically increased in recent years from just 0.6% in 2013 to 3.6% in 2016 in the United States (1), 0.8% in 2013 to 4.7% in 2016 in England and Wales (2), and 5.4% in 2013 to 7.1% in 2015 across Europe (3). Additionally, reports in 2015 from both China and Japan have documented resistance in as high as 30% of the gonococcal population in some regions (4). This spike in resistance is alarming, as azithromycin is one of the two first-line drugs, in conjunction with ceftriaxone, recommended as dual-antimicrobial therapy for uncomplicated cases of gonococcal infection by the Centers for Disease Control and Prevention (CDC) (1, 5). Azithromycin is a macrolide antibiotic that inhibits protein synthesis by binding to the 23S rRNA component of the 50S ribosome, and while the majority of resistance can be explained by mutations in the 23S rRNA azithromycin binding sites (C2611T and A2059G) (6–8), the genetic basis of a large fraction of resistance in the U.S. population is still unexplained (6), thus limiting the potential for development of molecular biology-based resistance diagnostics and restricting our understanding of the evolutionary paths to resistance.

Gonococci are adept at acquiring chromosomally encoded antimicrobial resistance determinants as a result of their natural competence for transformation, allowing for the spread of resistance and other adaptively advantageous alleles between lineages and even across species boundaries (9–12). Extensive intragenus gene exchange has led to the concept of *Neisseria* as a consortium of species interconnected by allele sharing, with “fuzzy” borders permitting rapid access to new adaptive solutions (12, 13). This produces genetic mosaicism within particular lineages, whereby some loci are the products of horizontal gene transfer and homologous recombination from other species. In gonococci, intragenus recombination is an important source of novel genetic variation with many observations of mosaic loci gained from other *Neisseria* species (14–18). However, aside from horizontal gene transfer facilitating the evolution of resistance to third-generation cephalosporins through acquisition of mosaic *penA* (8, 15), allelic exchange has not yet been demonstrated to be the basis for resistance to any other antibiotic class in this species.

Recent epidemiological studies from the United States, Canada, and Australia have reported an association between mosaic multiple transferable resistance (*mtr*) efflux pump alleles and increased resistance to azithromycin (8, 18–20). Mosaics at *mtr* appear to have originated through horizontal gene exchange from other *Neisseria* and have been identified by high sequence homology of the repressor of the pump (MtrR) to Neisseria meningitidis and Neisseria lactamica. Mosaic *mtr* alleles have previously been associated with an outbreak of azithromycin resistance in Kansas City, Missouri, from 1999 to 2000 (21, 22), and with the majority of azithromycin resistance reported in New South Wales, Australia (20). While correlation between mosaic *mtr* and azithromycin resistance suggests causality, there is little experimental evidence to confirm the association.

The Mtr efflux pump is comprised of the MtrC-MtrD-MtrE cell envelope proteins, which together export diverse hydrophobic antimicrobial agents such as antibiotics, nonionic detergents, antibacterial peptides, bile salts, and gonadal steroidal hormones from the cell (23–27). Overexpression of *mtrCDE* has been linked to Mtr-mediated resistance to diverse antimicrobial agents through increased drug export and also higher *in vivo* fitness in the murine genital tract (24, 28). Known mutations that alter expression of the pump include the *mtrC*_120_ substitution, an adenine-to-guanine transition located 120 bp upstream of the *mtrC* start codon which acts as an alternative promoter for *mtrCDE* (29); deletion of a single adenine (A deletion) in the *mtrCDE* promoter that has been shown to repress the transcription of *mtrR* while simultaneously enhancing transcription of *mtrCDE* (30); and mutations that abrogate the function of MtrR by inducing premature stop codons or radical amino acid substitutions in the DNA-binding motif (21, 31, 32). Finally, the presence or absence of a Correia element with an integration host factor (IHF) binding site inserted between *mtrCDE* and the *mtrCDE* promoter has also been shown to negatively impact *mtrCDE* transcription in N. meningitidis, perhaps through the formation of DNA secondary structure, which could act as a weak transcriptional terminator (33). However, it is unclear if resistance in *mtr* mosaics is derived from any of these mechanisms and alteration of *mtrCDE* transcription.

Here, we used a combination of population genomic and experimental approaches to dissect the mechanism of resistance in mosaics. We first assessed patterns of allelic diversity within the gonococcal population to define the boundaries of horizontal gene transfer at *mtrRCDE* and found that the entire *mtr* region is a hot spot of interspecies recombination which has introduced multiple rare and divergent mosaic alleles, likely from N. meningitidis and N. lactamica, into the gonococcal population. Strong linkage disequilibrium at *mtrD* and the *mtr* promoter region suggested the maintenance of epistatic allelic combinations; therefore, we tested for interaction effects within and between *mtr* loci via transformation. We discovered epistatic interactions across almost the entire *mtrD* gene, and also between mosaic *mtrD* and mosaic *mtr* promoter regions, that synergistically increased resistance to azithromycin and other substrates of the Mtr efflux pump. Furthermore, patterns of nucleotide diversity in this region coupled with experimental *in vitro* evidence suggest antibiotic-mediated selection may be acting to maintain these epistatic interactions at the population level in nature. Finally, we tested for regulatory evolution of pump components, as previous mechanisms of azithromycin resistance through the Mtr efflux pump have been demonstrated to be driven by expression. Our results support that inheritance of mosaic promoter regions increases the expression of *mtrCDE* while gaining mosaic *mtrD* alone does not. Thus, the likely mechanism of resistance in mosaics is a structural change to MtrD, which enhances the capacity of the protein to recognize or transport diverse antimicrobial agents, coupled with increased efflux through the amplified production of pump components.

## RESULTS

### Allelic diversity suggests increased interspecies admixture at *mtrRCDE.*

**FIG 1:**
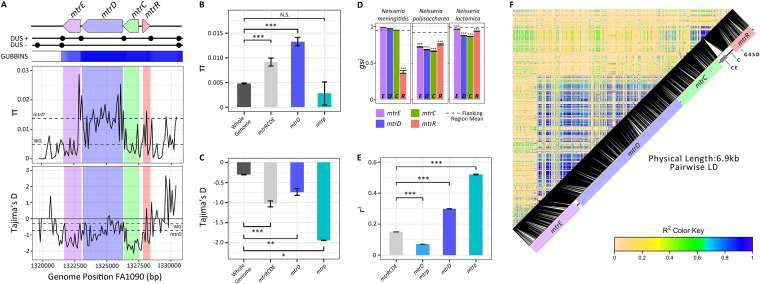
Horizontal gene transfer (HGT) of *mtr* introduces novel adaptive genetic variation into Neisseria gonorrhoeae. (A) The FA1090 genomic position of the *mtrRCDE* locus is presented, with the locations of DNA uptake sequences (DUS) displayed (black circles). These DUS sequences are shared with closely related *Neisseria* species and increase the frequency of DNA uptake and recombination (58). The U.S. gonococcal population (*n* = 1,102 isolates) shows patterns of elevated allelic diversity as measured by π and elevated SNP densities as predicted by Gubbins (52) across *mtrRCDE*. Depressed values of Tajima’s D (Tajima’s D < 0) indicate an excess frequency of rare mutations across this region. The average values for these parameters are plotted for *mtrD* (blue dashed lines) and the genome-wide average (gray dashed lines). (B) The highest allelic diversity within *mtrRCDE* was found at *mtrD* and (C) an excess of rare alleles was detected across *mtrRCDE* (Tajima’s D < 0) compared to the rest of the genome. The values that are significantly different are indicated by a bar and asterisks as follows: *, *P* less than or equal to 0.05; **, *P* less than or equal to 0.001; ***, *P* less than or equal to 0.0001. Values that are not significantly different (N.S.) are indicated. *mtrp*, *mtr* promoter. (D) Depressed genealogical sorting index (*gsi*) values compared to those of a 25-kb *mtr* flanking region indicate extensive admixture of alleles between N. gonorrhoeae and other *Neisseria* across all *mtr* loci. Signatures of high allelic diversity, an excess of rare mutations, and interspecies admixture taken altogether suggest the recent introduction of novel genetic variation into this region. (E) Linkage disequilibrium was also observed, with the highest linkage measured by *r*^2^ occurring within *mtrD* and the *mtr* promoter regions. (F) *r*^2^ values plotted across *mtrCDE* in reference to their position in strain FA1090 are represented from 0 (yellow for low linkage) to 1 (blue for high linkage). Positions of the Correia element insertion (CE), A-to-C transversion in the *mtrR* promoter inverted repeat (C), and glycine to aspartic acid MtrR amino acid substitution (G45D) are indicated. Large tracts of high linkage suggest recent recombination of alleles from another source or maintenance of particular haplotypes due to selection. LD, linkage disequilibrium.

To gain insight into the evolutionary history of the *mtrR* transcriptional repressor and the *mtrCDE* pump, we analyzed patterns of diversity using the 1,102 Gonococcal Isolate Surveillance Project (GISP) isolates described by Grad et al. (8, 34). A significant increase in allelic diversity was observed across *mtrRCDE* compared to the entire genome, with the highest diversity at *mtrD* (Fig. 1A and B; see Table S1 in the supplemental material). We also detected a significant enrichment of rare alleles in the population across *mtrRCDE* (Fig. 1A and C; Table S1). Linkage disequilibrium was strongest at *mtrD* and the *mtr* promoter region in a comparison of all pairs of single-nucleotide polymorphisms (SNPs) within *mtrRCDE*, with the highest linkage observed at pairs of variant sites within each of these loci (Fig. 1E and F; Table S1).

10.1128/mBio.01419-18.2 TABLE S1  Population genetics statistics calculated for all *mtr* loci. Download TABLE S1, XLSX file, 0.04 MB. Copyright © 2018 Wadsworth et al. 2018 Wadsworth et al. This content is distributed under the terms of the Creative Commons Attribution 4.0 International license.

To define interspecific admixture events within *Neisseria*, we characterized the genealogical sorting index (*gsi* [35]) to explore gene tree topology measures of species-specific phylogenetic exclusivity. *gsi* ranges from 0 (no exclusivity) to 1 (monophyletic) and serves as a metric to assess allele sharing that may arise through interspecific recombination or incomplete divergence from a recent split between species. We calculated *gsi* values for genes in a 25-kb window encompassing *mtrRCDE* with conserved microsynteny between N. gonorrhoeae and other *Neisseria* species (see Fig. S1 in the supplemental material). This region included 29 core genes excluding *mtrRCDE*. To define the region-specific *gsi* background, we calculated *gsi* values across 100 bootstrap replicates for each gene in the *mtrRCDE* flanking region by species. The mean gonococcal *gsi* values for flanking genes were 0.95 for N. meningitidis, 0.99 for N. lactamica, and 0.92 for Neisseria polysaccharea (Fig. 1D; Table S2). Significant reductions in *gsi* values were detected across *mtrRCDE* compared to the 29 loci within the surrounding 25-kb region (Fig. 1D). Significant allele sharing between N. gonorrhoeae and N. meningitidis was exclusively observed at *mtrR* (Fig. 1D; Table S2), while significant allele sharing between N. gonorrhoeae and both N. lactamica and N. polysaccharea occurred across all *mtr* loci (Fig. 1D; Table S2).

10.1128/mBio.01419-18.1 FIG S1  Fully sequenced, annotated genomes for N. gonorrhoeae (top), N. lactamica (middle), and N. meningitidis (bottom) were aligned with progressiveMauve (54). Syntenic blocks between these three species are illustrated by colored rectangles. For instance, the purple rectangles above the red arrows show a large (~100-kb) collinear genomic segment shared among the three *Neisserial* species. This syntenic block contains the *mtr* operon (positions 1,323,095 to 1,326,298). Download FIG S1, TIF file, 79.5 MB. Copyright © 2018 Wadsworth et al. 2018 Wadsworth et al. This content is distributed under the terms of the Creative Commons Attribution 4.0 International license.

10.1128/mBio.01419-18.3 TABLE S2  Genealogical sorting index (*gsi*) values as a measure of genealogical exclusivity and admixture. Download TABLE S2, XLSX file, 0.04 MB. Copyright © 2018 Wadsworth et al. 2018 Wadsworth et al. This content is distributed under the terms of the Creative Commons Attribution 4.0 International license.

**FIG 2:**
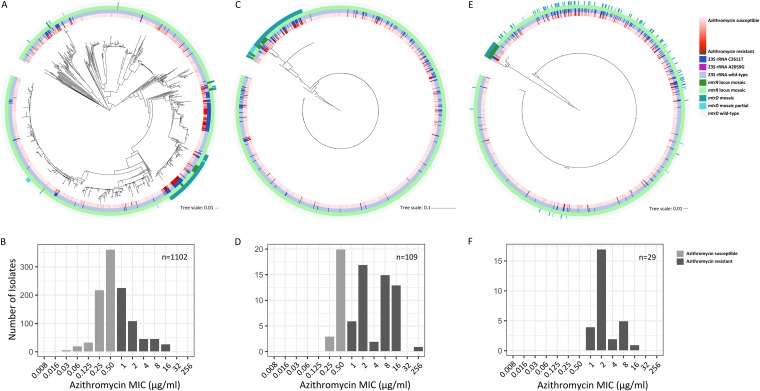
Divergent *mtrD* and *mtr* promoter haplotypes are associated with increases in azithromycin MIC. (A) A maximum likelihood whole-genome-sequence phylogeny of 1,102 Neisseria gonorrhoeae isolates based on single-nucleotide polymorphisms generated from mapping to the FA1090 reference genome (8). The inner annotation ring shows azithromycin MICs on a continuous scale (red shading). The next annotation ring indicates isolates with at least two copies of the C2611T 23S rRNA mutation (dark blue) or isolates with four copies of the A2059G 23S rRNA mutation (magenta). The next annotation ring shows isolates that were identified as interspecies mosaics based on their sequence at *mtrR* by Grad et al. (8) (dark green). The outermost annotation ring shows isolates identified as *mtrD* mosaics in this study (teal). Tree scales or bars, 0.01 or 0.1 nucleotide substitutions per position. (B) The entire gonococcal population has a distribution of azithromycin MIC values which fall both above and below the defined resistance threshold (MIC ≥ 1 µg/ml). (C) A maximum likelihood phylogeny built on *mtrD* alignments shows 109 isolates with full-length mosaic alleles that are highly divergent from the wild-type gonococcal *mtrD* allele. (D) These 109 isolates are associated with elevated azithromycin MICs (MICs ≥ 0.25 µg/ml). (E) A maximum likelihood phylogeny built on the *mtr* promoter region shows that all 29 of the *mtr* promoter mosaics previously described by Grad et al. (8) also have inherited mosaic *mtrD*. (F) Isolates with both full-length mosaic *mtrD* and a mosaic *mtr* promoter region alleles have higher azithromycin MICs (MICs ≥ 1 µg/ml) than those isolates with mosaic *mtrD* alone.

There were multiple recombined mosaic haplotypes present in the gonococcal population carrying the full-length *mtrD* (*n* = 80), *mtrRCD* (*n* = 9), and *mtrRCDE* (*n* = 20) genes and some isolates with partial mosaic *mtrD* with the majority of the gene homologous to native gonococcal sequence (*n* = 13) (Fig. 2). Of the 109 isolates with full-length mosaic *mtrD*, 4 isolates had mosaic *mtrD* genes that were 99% identical to the N. meningitidis gene, 5 isolates had mosaic *mtrD* alleles with 94 to 96% identity to the N. lactamica gene, and the remainder had alleles that aligned equally well to N. meningitidis and N. lactamica with identities ranging from 91 to 92%. Of the 29 isolates with mosaic promoter regions identified by Grad et al. (8), 24 isolates had mosaic promoter regions that were 96 to 98% identical to those in N. lactamica, 4 had regions that were 99% identical to the N. meningitidis region with the presence of a 153-bp Correia element insertion (21), and 1 isolate had a region that was 92% similar to the N. meningitidis region but lacked the Correia element that was present in the other 4 N. meningitidis-like isolates. While we find the highest sequence similarity of mosaic sequences to N. meningitidis and N. lactamica in the NCBI database, the paucity of sequences representing commensal *Neisseria* presents an issue with conclusive species annotation. However, it is clear that mosaic alleles are highly divergent from the native gonococcus-like alleles, which are relatively monomorphic. All isolates with full-length mosaic *mtrD* had azithromycin MICs of ≥0.25 µg/ml, while all isolates with full-length mosaic *mtrD* and a mosaic *mtr* promoter had azithromycin MICs of ≥1 µg/ml (Fig. 2).

Of the 29 *mtr* mosaics described by Grad et al. (8) and identified by a mosaic *mtr* promoter sequence, none had the *mtrC*_120_ substitution, A deletion, 23S rRNA mutation A2059G, mutations in *rplD*, *rplV* tandem duplications, or variants of the rRNA methylase genes *ermC* and *ermB* that have been associated with or experimentally confirmed to be involved in azithromycin resistance (7, 8, 19, 24, 29–32). However, four mosaic isolates had the premature stop codon mutations in *mtrR*, and five had the C2611T 23S rRNA mutation (6). While most of the isolates with full-length mosaic *mtrD* and a mosaic *mtr* promoter had azithromycin MICs ranging from 1 to 4 µg/ml, those with the *mtrR* premature stop codon or the C2611T 23S rRNA mutation had higher MICs ranging from 8 to 16 µg/ml, most likely due to the additive or epistatic effects of inheriting multiple mechanisms of resistance.

### Epistasis between multiple *mtr* loci and within *mtrD.*

We exploited the natural competence of *Neisseria* to explore the potential for mosaic *mtr* alleles to produce an increase in resistance to azithromycin by transforming susceptible strains with either genomic DNA (gDNA) or PCR-amplified products from mosaic donors. Susceptible recipient strains for transformations included 28Bl (21, 36, 37), GCGS0353, and GCGS0465 (MIC ≤ 0.125 µg/ml; Table 1). Three strains with reported mosaic *mtr* alleles and azithromycin MICs of ≥1 µg/ml were selected as donors for DNA transfer (Table 1). These isolates included GCGS0276, GCGS0834, and GCGS0402. Of these mosaics, GCGS0276 had an *mtrR* sequence with the highest percent sequence identity to N. meningitidis isolates in the NCBI database, while GCGS0834 and GCGS0402 had *mtrR* genes most similar to N. lactamica isolates. Thus, we refer to these isolates as N. meningitidis-like and N. lactamica-like *mtr* mosaics, respectively. None of the selected donor strains had premature stop codons in *mtrR* or the C2611T mutation.

**TABLE 1:** Properties of strains used in the study

Strain	Source^a^	MIC (µg/ml)^b^			
		AZI	CV	PB	TX-100
GCGS0276	GISP isolate; Kansas City, MO	1	8	1,000	16,000
GCGS0402	GISP isolate; Miami, FL	2	8	500	>16,000
GCGS0834	GISP isolate; Los Angeles, CA	2	8	500	>16,000
GCGS0353	GISP isolate; Dallas, TX	0.03	1	60	<250
GCGS0465	GISP isolate; Phoenix, AZ	0.06	0.5	125	<250
28Bl	Disseminated gonococcal infection isolate (CDC, 1974)	0.125	4	60	<250

a GISP isolates are from the Gonococcal Isolate Surveillance Project. The geographical location where the specimen was collected is shown.

b AZI, azithromycin; CV, crystal violet; PB, polymyxin B; TX-100, Triton X-100. MIC values are the modes from three independent replicate tests.

**FIG 3:**
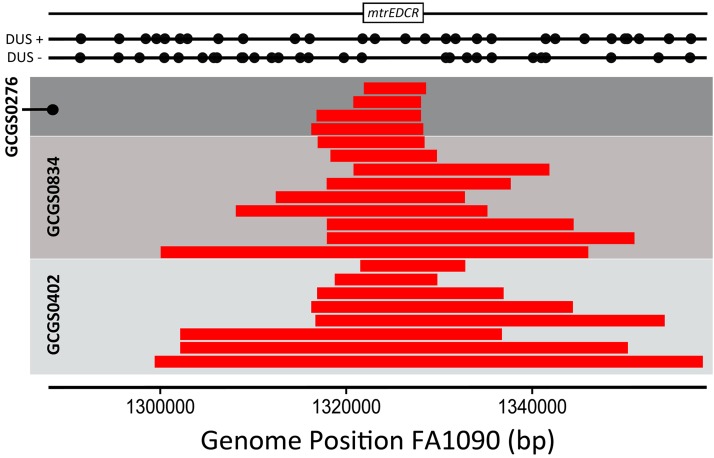
Unguided genomic DNA transformations demonstrate that the mosaic *mtrRCDE* region causes increased azithromycin resistance. The 28Bl azithromycin-susceptible recipient strain was transformed with gDNA from mosaic *mtr* donor strains and selected on plates containing 0.38 to 1 µg/ml azithromycin. Genomic sequencing revealed the boundaries of recombined DNA that azithromycin-resistant transformants had inherited from mosaic donor strains (red bars) in the 28Bl background, as identified by SNP homology using the FA1090 reference genome as a scaffold. The only genomic region that was consistently inherited from donor strains in the transformant cell lines encompassed *mtrRCDE* for both the N. meningitidis (GCGS0276) and N. lactamica-like (GCGS0402 and GCGS0834) *mtr* mosaics. Average *in vitro* recombination tract lengths were 9.5 ± 1.4 kb for strain GCGS0276 (*n* = 4), 24.3 ± 3.6 kb for GCGS0834 (*n* = 9), and 31.4 ± 6.0 kb for GCGS0402 (*n* = 8). The locations of all DNA uptake sequences (DUS) (black circles), as well as the *mtr* locus (white box), are mapped in reference to the FA1090 strain.

Genomic DNA from *Neisseria* GCGS0276, GCGS0402, and GCGS0834 donors was able to transform multiple susceptible recipient isolates to resistance on selective media containing azithromycin (Table 2, genomic DNA transformant strains). To identify the locus responsible, we sequenced the genomes of 28Bl cell lines transformed with gDNA from mosaic donors and characterized SNPs that had been inherited from donor strains that were not present in the 28Bl recipient. The only common region that had been inherited across all transformants contained the *mtrRCDE* locus (Fig. 3). Likewise, the susceptible GCGS0353 and GCGS0465 cell lines transformed with gDNA from mosaic donors and subsequently selected for resistance to azithromycin were also confirmed to have inherited mosaic *mtrD* and *mtr* promoter alleles (Table 2, genomic DNA transformant strains). Thus, we further characterized this region using both population genomic and experimental approaches to carefully detail the genetic basis of resistance at *mtr* in mosaic isolates.

**TABLE 2:** MIC values of transformant strains

Strain	Recipient strain	Transformation substrate	MIC (µg/ml)^a^				Mosaic *mtrD*^b^	Mosaic *mtrp*^b^
			AZI	CV	PB	TX-100		
Genomic DNA (gDNA) transformant strains								
28BlΔGCGS0276-gDNA	28Bl	GCGS0276 gDNA	2	16	500	>16,000	+	+
GCGS0353ΔGCGS0276-gDNA	GCGS0353	GCGS0276 gDNA	1	16	1,000	>16,000	+	+
GCGS0465ΔGCGS0276-gDNA	GCGS0465	GCGS0276 gDNA	2	16	500	>16,000	+	+
28BlΔGCGS0402-gDNA	28Bl	GCGS0402 gDNA	2	16	500	>16,000	+	+
GCGS0353ΔGCGS0402-gDNA	GCGS0353	GCGS0402 gDNA	2	16	500	>16,000	+	+
GCGS0465ΔGCGS0402-gDNA	GCGS0465	GCGS0402 gDNA	2	16	500	>16,000	+	+
28BlΔGCGS0834-gDNA	28Bl	GCGS0834 gDNA	2	16	500	>16,000	+	+
GCGS0353ΔGCGS0834-gDNA	GCGS0353	GCGS0834 gDNA	2	16	500	>16,000	+	+
GCGS0465ΔGCGS0834-gDNA	GCGS0465	GCGS0834 gDNA	2	16	500	>16,000	+	+

PCR product transformant strains								
28BlΔGCGS0276-mtrRpCDE	28Bl	GCGS0276 *mtrRpCDE*	1	16	500	>16,000	+	+
28BlΔGCGS0276-mtrD	28Bl	GCGS0276 partial *mtrC* (1185–1241 bp) and *mtrD* (1–3174 bp)	0.5	8	250	>16,000	+	−
28BlΔGCGS0276-mtrD/18-3174	28Bl	GCGS0276 partial *mtrD* (18–3174 bp)	0.5	8	250	16,000	+	−
28BlΔGCGS0276-mtrD/+262-2724	28Bl	GCGS0276 partial *mtrC* (1185–1241 bp) and *mtrD* (1–2724 bp)	0.5	8	250	>16,000	+	−
28BlΔGCGS0276-mtrDCp	28Bl	GCGS0276 *mtrD*, *mtrC*, *mtr* promoter	1	16	500	>16,000	+	+
28BlΔGCGS0402-mtrRpCDE	28Bl	GCGS0402 *mtrRpCDE*	2	16	500	>16,000	+	+
28BlΔGCGS0402-mtrDCp	28Bl	GCGS0402 *mtrD*, *mtrC*, *mtr* promoter	2	16	500	>16,000	+	+

a AZI, azithromycin; CV, crystal violet; PB, polymyxin B; TX-100, Triton X-100. MIC values are the modes from three independent replicate tests.

b The presence (+) of mosaic alleles was screened for by Sanger or whole-genome sequencing.

**FIG 4:**
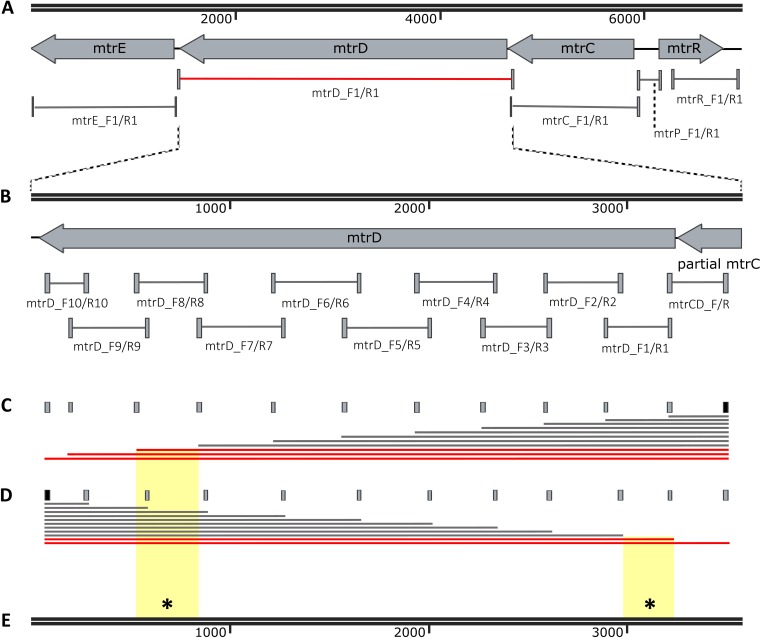
Epistatic interactions between multiple domains of *mtrD* contribute to elevated azithromycin MICs. (A) GCGS0276 *mtrD* in the 28Bl background elevated the azithromycin MIC from 0.125 µg/ml to 0.5 µg/ml (red). (B) Primer pairs designed to amplify ~300-bp fragments over the length of *mtrD* (see Table S4) resulted in no observed transformants on 0.38-µg/ml azithromycin selection plates, suggesting that multiple mutations across *mtrD* are needed for resistance. (C and D) To determine the regions that contributed to resistance, multiple fragment sizes were constructed by holding the rightmost forward primer (black) constant while adding different reverse primers (gray) (C) and by holding the leftmost reverse primer (black) constant while adding forward primers (gray) to separate reactions (D). The red lines in panels C and D indicate the PCR products that generated transformants on azithromycin selection plates. (E) At a minimum, SNPs at base pair positions 18 to 356 coupled with SNPs at positions 2356 to 2724 (indicated by yellow background and an asterisk) were required to raise the MIC from the 0.125 µg/ml of the recipient 28Bl strain to 0.5 µg/ml, though we are unable to exclude the possibility that additional SNPs between these two regions are also involved.

**FIG 5:**
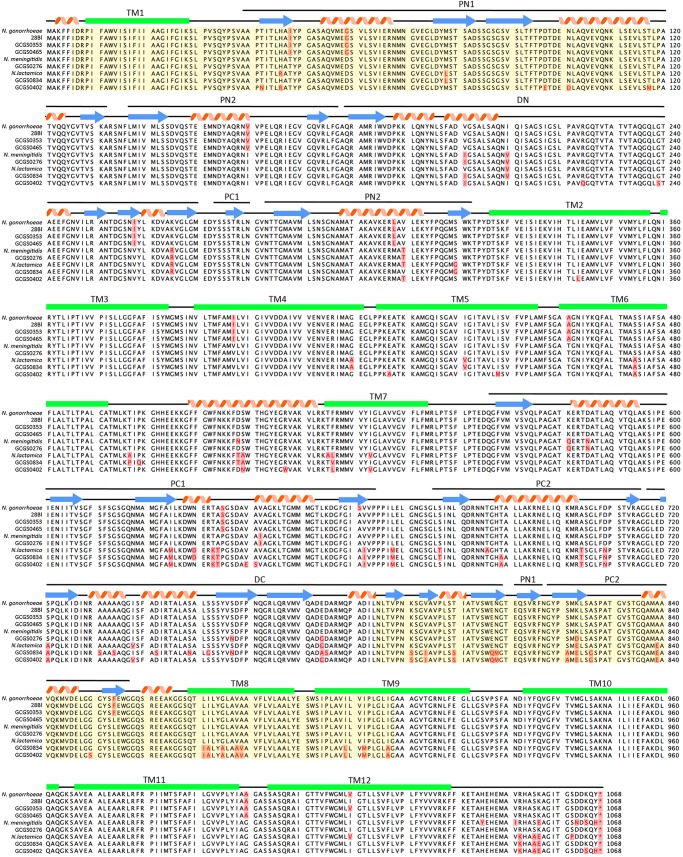
Alignment of wild-type and mosaic Neisseria gonorrhoeae MtrD with N. lactamica and N. meningitidis reference sequences. Amino acid alignment of MtrD sequences from N. gonorrhoeae FA1090 (AE004969.1), N. lactamica 020-06 (FN995097.1), and N. meningitidis NZ-05/33 (CP002424.1) with those of the gonococcal isolates used in this study are shown. The minor variants for each amino acid position are shown in red on a pink background. Though most of the amino acid changes in isolates with mosaic MtrDs are homologous to those found in either the chosen N. lactamica and N. meningitidis references, some amino acid changes are unique and thus have been either horizontally acquired from another source or represent *de novo* mutations. Overlaid on the alignment are the secondary structure elements of the protein: β pleated sheets (blue arrows); α-helices (red spirals); and transmembrane helices (TM) (green boxes) (40). Domains of MtrD are annotated and include the MtrE docking domain (DN and DC) and the MtrD pore domain (PN1, PN2, PC1, and PC2) (40). Epistatically interacting domains of MtrD that enhanced azithromycin MIC in mosaic isolates as deduced from transformation experiments are shown on yellow background.

Primers were designed to amplify each locus in *mtr* for both a N. meningitidis-like mosaic (GCGS0276) and a N. lactamica-like mosaic (GCGS0402), and the resultant amplicons were then used to transform strain 28Bl (Fig. 4A; Table S3). For strain GCGS0276, the only locus within the *mtrRCDE* operon that was found to increase resistance to azithromycin independently was *mtrD* (Fig. 4A; Table 2, PCR product transformant strains). GCGS0276 *mtrD* in the 28Bl background raised the azithromycin MIC threefold from 0.125 to 0.5 µg/ml, yet no single region of *mtrD* was able to produce the 0.5 µg/ml phenotype independently (Fig. 4B). Population genomics results indicated the presence of high linkage disequilibrium across *mtrD* (Fig. 1E and F). Thus, to test for possible within-gene epistatic interaction effects, we designed a series of amplicons that incorporated increasingly larger fragments of the *mtrD* locus (Fig. 4C and D). Amplicons that contained both the 5′ end (18 to 356 bp) and 3′ end (2356 to 2724 bp) of GCGS0276 *mtrD* were together sufficient to increase the 28Bl azithromycin MIC to 0.5 µg/ml (Fig. 4E). There were four changes at the amino acid level between GCGS0276 and 28Bl in these regions, two in the PN1 domain of MtrD (I48T and G59D) and two in the PC2 domain (K823D and F854L) (Fig. 5), and in total, 20 amino acid changes between the GCGS0276 and 28Bl proteins (Fig. 5). Unlike the N. meningitidis-like *mtrD* allele from GCGS0276, the N. lactamica-like GCGS0402 *mtrD* allele was not able to produce resistance to azithromycin in the 28Bl background above the 0.38-µg/ml-concentration selection plates.

10.1128/mBio.01419-18.4 TABLE S3  Oligonucleotide primers and PCR conditions used for construction of the DNA templates for transformation and Sanger sequencing to confirm transformation. Download TABLE S3, XLSX file, 0.01 MB. Copyright © 2018 Wadsworth et al. 2018 Wadsworth et al. This content is distributed under the terms of the Creative Commons Attribution 4.0 International license.

**FIG 6:**
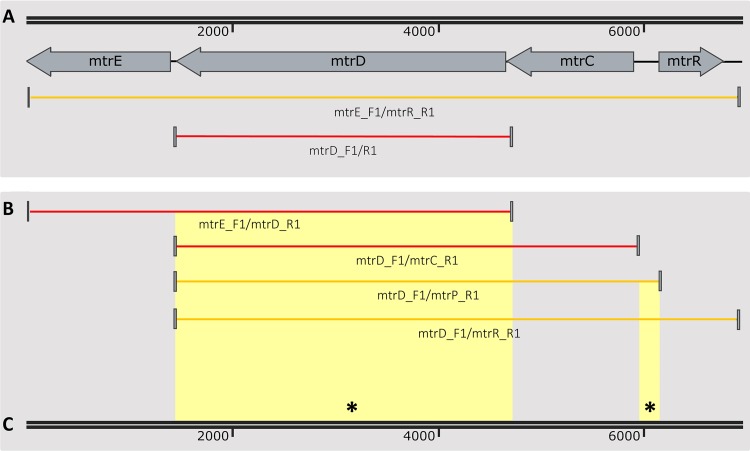
Epistasis between *mtrD* and the *mtr* promoter region yields increased azithromycin resistance in mosaic isolates. (A) While transformation of the entire GCGS0276 *mtrRCDE* region perfectly reconstructed the donor strain MIC of 1 µg/ml in the 28Bl background (yellow line), GCGS0276 *mtrD* was the only region within *mtrRCDE* that could independently increase resistance to azithromycin (from 0.125 to 0.5 µg/ml [red line]). This suggested that epistasis across loci contributed to resistance in *mtr* mosaics. (B) To determine the additional locus needed to produce the high-level resistance seen in *mtr* mosaics, PCR products were designed to amplify *mtrD* in addition to other loci within *mtrRCDE*. Here, we found that the addition of the *mtr* promoter to constructs containing *mtrD* was sufficient to raise the MIC of 28Bl to the GCGS0276 phenotype of 1 µg/ml.

**FIG 7:**
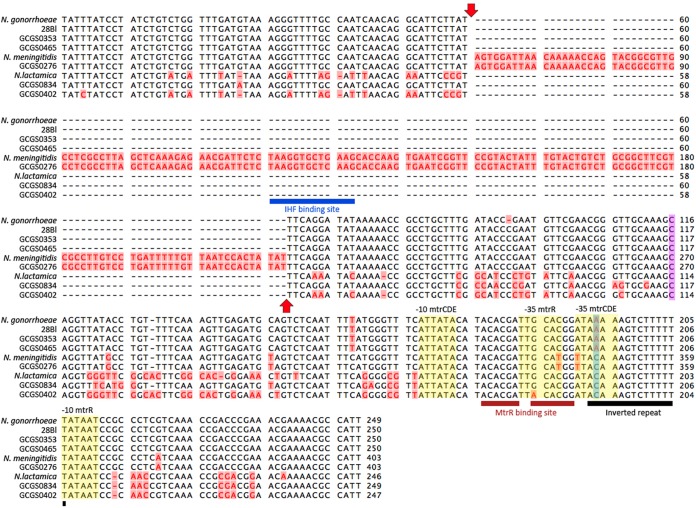
Alignment of wild-type and mosaic Neisseria gonorrhoeae *mtr* promoters with N. lactamica and N. meningitidis references. Nucleotide alignment of the *mtr* promoter region from N. gonorrhoeae FA1090 (AE004969.1), N. lactamica 020-06 (FN995097.1), and N. meningitidis NZ-05/33 (CP002424.1) with the gonococcal isolates used in this study. The minor allele variants for each position are shown in red on a pink background. Sequence features include the interleaved *mtrCDE* and *mtrR* promoters (yellow background), MtrR binding site (maroon bars), inverted repeat in the *mtrR* promoter (black bar), 153-bp Correia element insertion (red arrows), and integration host factor (IHF) binding site (blue bar) (21, 29, 30, 33). The C-to-T transition 120 bp upstream of the *mtrC* start codon (*mtr*_120_ [purple background]) has been demonstrated to increase expression of the efflux pump components and enhance resistance to various substrates of the pump (29); however, no T variants were detected at this site. Deletion of a single A (A deletion) in the *mtrR* promoter inverted repeated (blue background) has also been shown to increase expression of the efflux pump and enhance *mtr*-mediated resistance (30); no A-deletion variants were present. Interestingly, the N. lactamica reference, N. meningitidis reference, and all 29 isolates with mosaic *mtr* promoter regions had a C transversion in the second position within this repeat (blue background).

Though the mosaic GCGS0276 *mtrD* allele was clearly instrumental in enhancing resistance to azithromycin in strain 28Bl, it was not sufficient on its own to reproduce the level of resistance observed in the GCGS0276 donor strain. However, transformants that inherited the entire *mtrRCDE* operons of strains GCGS0276 and GCGS0402 had azithromycin MICs of 1 and 2 µg/ml, respectively, perfectly mirroring the donor strain resistance phenotypes (Table 2; Fig. 6A). Thus, in order to determine whether between-gene epistatic interactions resulted in donor strain resistance, amplicons were designed for each of these strains to amplify the *mtrD* locus in combination with other regions of the operon. Using this strategy, we found that for both strains GCGS0276 and GCGS0402, the donor strain azithromycin MICs of 1 and 2 µg/ml could be reproduced in strain 28Bl by transforming both *mtrD* and the *mtr* promoter region together (Fig. 6B; Table 2, PCR product transformant strains). Multiple mutations were present between isolates with mosaic and wild-type gonococcal *mtr* promoters; however, the only conserved mutation in all 29 mosaics was an A-to-C transversion in the *mtrR* promoter inverted repeat (Fig. 7).

### Mosaic *mtr* mutations contribute to resistance to diverse antimicrobial agents.

The Mtr efflux pump exports diverse antimicrobial agents from the cell (23–27). To determine whether mosaic *mtr* mutations were specific to enhancing azithromycin recognition or transport, or were capable of enhancing resistance to multiple substrates of the pump, we tested the susceptibilities of all strains and transformant lines to the dye crystal violet and the detergents Triton X-100 and polymyxin B.

All azithromycin-resistant mosaic *mtr* isolates had higher MICs for the additional antimicrobials tested than the azithromycin-susceptible GCGS0353, GCGS0465, and 28Bl isolates (Table 1). Genomic DNA transformants screened for the presence of both a mosaic *mtrD* and a mosaic *mtr* promoter region from either N. meningitidis-like or N. lactamica-like donors and transformants with mosaic *mtrRCDE* or *mtrpCD* from GCGS0276 or GCGS0402 had elevated MICs for these drugs compared to susceptible recipient strains (Table 2). GCGS0276 *mtrD* in the 28Bl genomic background increased MICs for crystal violet and polymyxin B by twofold (from 4 µg/ml to 8 µg/ml) and fourfold (from 60 µg/ml to 250 µg/ml), respectively, while addition of the mosaic *mtr* promoter increased MICs 1 dilution higher in both cases (Table 2).

### Regulatory and structural mutations epistatically contribute to resistance.

We tested for the contribution of transcript regulatory variation to the mechanism of resistance by profiling gene expression via transcriptome sequencing (RNA-seq) of strains 28Bl, 28BlΔGCGS0276-mtrD, and 28BlΔGCGS0276-mtrRpCDE. As expression of the *mtr* efflux pump is inducible by exposure to antimicrobial agents (38, 39), we evaluated expression before azithromycin exposure and 120 min after the addition of a sub-MIC dose of azithromycin (0.125 µg/ml) to the culture media. Across 24 libraries, a total of 106 million 50-bp paired-end reads mapped to the FA1090 reference genome. Each library had on average 4.44 ± 3.49 million mappable reads.

**FIG 8:**
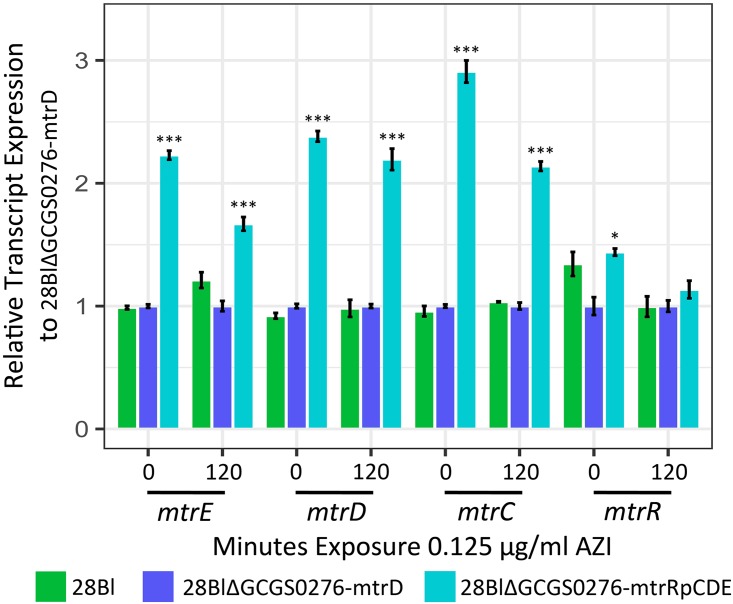
The N. meningitidis-like GCGS0276 mosaic *mtr* promoter sequence upregulates expression of *mtr* efflux pump component mRNAs. The 28Bl recipient strain (green), 28Bl transformants with mosaic GCGS0276 *mtrD* (blue), and 28Bl transformants with mosaic GCGS0276 *mtrRCDE* (teal) were exposed to sub-MIC (0.125 µg/ml) concentrations of azithromycin (AZI) for 120 min and subjected to whole-transcriptome sequencing before and after the addition of the antimicrobial. The 28BlΔGCGS0276-mtrD strain was then used as a reference to calculate the relative expression of transcripts for other strains by condition by gene. In both the presence and absence of drug, transformants with the presence of a mosaic *mtr* promoter region yielded significantly upregulated pump component mRNAs (false-discovery rate [FDR] of <0.0001), while transformants that inherited mosaic *mtrD* alleles did not significantly alter *mtrCDE* regulation from the 28Bl recipient strain levels. Values that are significantly different are indicated by asterisks as follows: *, *P* less than or equal to 0.05; *** *P* less than or equal to 0.0001.

We assessed the impact of mosaic *mtrD* on *mtrRCDE* mRNA expression by comparing 28BlΔGCGS0276-mtrD transformants to 28Bl and found no significant differential regulation of transcripts encoding *mtr* efflux pump components (Fig. 8; Table S4). To determine the effect of the mosaic *mtr* promoter on pump expression, we compared 28BlΔGCGS0276-mtrD and 28BlΔGCGS0276-mtrRpCDE transformants (Fig. 8; Table S4). Here, the presence of a mosaic *mtr* promoter resulted in the significant upregulation of *mtrC*, *mtrD*, and *mtrE* across conditions (false-discovery rate [FDR] of <0.0001) and upregulation of *mtrR* in the absence of azithromycin (FDR of 0.003).

10.1128/mBio.01419-18.5 TABLE S4  Number of significantly differentially expressed (DE) genomic features between strains at a FDR of <0.01. Download TABLE S4, XLSX file, 0.01 MB. Copyright © 2018 Wadsworth et al. 2018 Wadsworth et al. This content is distributed under the terms of the Creative Commons Attribution 4.0 International license.

## DISCUSSION

Genomic and surveillance efforts from across the globe have shown an association between mosaic *mtr* alleles and azithromycin resistance (8, 18–20), but the causal role of these alleles in generating resistance has been unclear. Mtr-mediated resistance to macrolides has been attributed exclusively to increased *mtrCDE* expression (28–32). However, a majority of *mtr* mosaic isolates in the population genomic data set have none of the variants that alter regulation of *mtrCDE*, such as the *mtrC*_120_ substitution, deletion of a single adenine (A deletion) in the *mtrR* promoter inverted repeat, radical *mtrR* amino acid changes, or premature stop codon mutations in *mtrR* (28–32). Thus, using a combination of experimental and population genomic approaches, we set out to better characterize the mechanism of resistance in isolates with mosaic *mtr* sequences.

Population genomics and phylogenetic reconstruction results support *mtrRCDE* as a hot spot of interspecies allele sharing. Expectations for genomic signatures of recent horizontal gene transfer events include the following: (i) local elevation of allelic diversity and an excess of rare mutations prior to the fixation, rise to intermediate frequencies, or loss of new alleles from the population; (ii) a departure from species-specific phylogenetic tree topology, indicating the admixture of alleles for particular loci; and (iii) high linkage within the recombination tract as the combined effects of recombination and mutation do not have sufficient evolutionary time to break down local associations between sites. Our results demonstrate that each of these parameters departed from the genomic background at *mtrRCDE*, suggesting recent horizontal gene transfer as opposed to historical admixture in introducing novel haplotypes at this locus (Fig. 1). While historical admixture can also produce patterns of polyphyly between species, the rarity of mosaic alleles and their high linkage suggests a more recent origin in the gonococcal population.

At least 12 independent acquisitions of mosaic *mtr* alleles are supported by a whole-genome-based phylogeny of the U.S. gonococcal population samples (Fig. 2A), which have introduced multiple rare and divergent *mtr* haplotypes from N. meningitidis and N. lactamica into the gonococcal population. Multiple mosaic haplotypes were present in the population, some spanning exclusively over *mtrD*, while others extended over both *mtrD* and the *mtr* promoter region (Fig. 2C and E). Isolates with mosaic sequences were associated with increased azithromycin MIC, where isolates with full-length mosaic *mtrD* sequences had MICs of ≥0.25 µg/ml (Fig. 2D) and isolates with both mosaic *mtrD* and *mtr* promoters had MICs of ≥1 µg/ml (Fig. 2F). These results mirror reports of isolates with mosaic *mtrR* or *mtr* promoter regions having azithromycin MICs of ≥1 µg/ml; however, prior studies did not screen for mosaic *mtrD* (8, 18–20). Interestingly, while admixture between N. gonorrhoeae and N. polysaccharea is also supported by depressed *gsi* values across *mtrRCDE*, we find no evidence of N. polysaccharea-like *mtr* alleles in the gonococcal population sample. This may suggest unidirectional transfer of N. gonorrhoeae *mtr* alleles into the N. polysaccharea population via horizontal gene transfer, or one ancestrally shared *mtr* allele in both species has risen to high frequency in the N. gonorrhoeae population.

Though mosaic *mtr* alleles have previously been associated with resistance (8, 18–20), our results conclusively demonstrate that they are responsible for resistance to diverse antimicrobial agents and show that the mechanism of resistance involves multiple synergistically acting epistatic interactions, including epistasis between multiple sites within *mtrD* and epistasis between MtrD and the *mtr* promoter region. Surprisingly, despite the sequence divergence (8%) between N. meningitidis- and N. lactamica-like *mtr* mosaics, *mtr* sequences from both generated azithromycin resistance through the same epistatic mechanism. For the N. meningitidis-like GCGS0276 mosaic, *mtrD* alone was capable of raising the azithromycin MIC of strain 28Bl to 0.5 µg/ml, independent of transcriptional changes to the pump’s regulation, with the donor azithromycin resistance phenotype of 1 µg/ml reproduced only by adding the N. meningitidis *mtr* promoter region, which increased expression of *mtrCDE* (Table 2; Fig. 4, 6, and 8). Similarly, although acquisition of the N. lactamica-like GCGS0402 *mtrD* was not sufficient on its own to enhance azithromycin resistance, transformation of both the *mtrD* and the *mtr* promoter region yielded the donor’s azithromycin MIC of 2 µg/ml (Table 2, PCR product transformant strains). All transformants that inherited mosaic *mtr* alleles in addition to becoming resistant to azithromycin also became resistant to diverse antimicrobials (Table 2). Thus, the mechanism of resistance in mosaics is likely derived from both structural changes to MtrD, which nonspecifically export antimicrobials more efficiently, coupled with promoter mutations that alter regulation of *mtrCDE*, enhancing drug export as a result of the increased availability of Mtr pump complexes.

Addition of the GCGS0276 mosaic *mtr* promoter region in the 28Bl background increased expression of *mtrCDE* (Fig. 8); however, this isolate did not have the *mtrC*_120_ substitution, A deletion, or mutations in *mtrR* that have been previously demonstrated to increase *mtrCDE* transcription (28–32). While we were unable to pinpoint the precise mutation(s) responsible for increased *mtrCDE* expression in this mosaic, two polymorphisms present in strain GCGS0276 have previously been implicated in altered *mtr* regulation. The GCGS0276 mosaic has a 153-bp Correia element inserted between *mtrC* and the *mtrCDE* promoter with an IHF binding site, which has been demonstrated in meningococci to decrease *mtrCDE* transcription, possibly by acting as a weak transcriptional terminator (33). As we show that the addition of the mosaic promoter sequence increases *mtrCDE* expression in N. gonorrhoeae, the Correia element is either (i) not having the same phenotypic effect in N. gonorrhoeae and N. meningitidis or (ii) decreases expression of *mtrCDE* as in N. meningitidis while another mutation increases the transcription of *mtrCDE* to an extent that the level of transcriptional termination caused by the Correia element has no significant impact. The second polymorphism, a conserved A-to-C transversion in the *mtrR* promoter inverted repeat, was present in all 29 isolates with mosaic *mtr* promoters (Fig. 7). Interestingly, this mutation is located in the same inverted repeat as an A deletion that has been shown to both increase transcription of *mtrCDE* and increase resistance to diverse antimicrobials (30). Additionally, a previously described isolate with an azithromycin MIC of 2 µg/ml was noted to have an A-to-C transversion of another site within this repeat (29).

Nearly the full-length *mtrD* was required for azithromycin resistance in GCGS0276 transformants, indicating the role of within-gene epistasis, rather than a single point mutation within *mtrD*, in generating resistance. Transformants that inherited two regions at the 5′ and 3′ ends of *mtrD* from strain GCGS0276 increased the azithromycin MIC of strain 28Bl to 0.5 µg/ml (Fig. 4). These two regions are part of the central pore (PN1) that stabilizes the trimeric organization of the protein and the outer periplasmic region (PC2) of the protein that may interact with MtrC to form a functional pump complex (40). Though we show that the 5′ and 3′ regions were both required to increase resistance in the 28Bl background, we are currently unable to exclude the possibility that additional SNPs between these two regions are involved in the interaction. Twenty additional amino acid changes were present between the GCGS0276 and 28Bl MtrD proteins (Fig. 5). Of note, none of the mutations observed in strains 28Bl and GCGS0276 have been shown to contribute to macrolide resistance in the orthologous proteins AcrB and MexB in other species, nor are they located in the direct contact site (residue 616) for macrolide recognition, the multidrug binding site of the pump (residues 608 to 619), or the sites involved in forming the proton relay network of the pump (40–44). Additional studies will be needed to determine the combination of mutations in *mtrD* responsible for enhanced resistance, though it is clear that whatever the causal SNPs are, they are acting to enhance resistance nonspecifically to multiple substrates of the pump (Table 2).

The selective maintenance of epistasis between domains of MtrD in natural populations of gonococci is supported by our experimental and genomics results. Across *mtrD*, we observed local increases in linkage disequilibrium coupled with an increased frequency of rare mutations (Fig. 1). These signatures could be explained by the recent acquisition of neutral diversity from closely related species, with too little time for the effects of recombination and mutation to break down linkage of sites across imported DNA tracts or the spread of these mutations to higher frequencies (45, 46). However, our experimental results demonstrate strong purifying selection on plates containing azithromycin after inheritance of partial *mtrD* mosaic haplotypes (Fig. 4), suggesting that some of the linkage within *mtrD* observed in natural gonococcal populations may be driven by selection maintaining allelic combinations within the gene that increase resistance to antimicrobials. We speculate that this is due to the effects of selection against partial gene recombination events between highly divergent *mtrD* alleles, which could produce defective lethal or inefficient protein products when under antimicrobial selection.

Overall, our results defining the role of mosaic *mtr* in azithromycin resistance affirm the importance of other *Neisseria* species as an antibiotic resistance reservoir for N. gonorrhoeae. Moreover, whereas mosaic *penA* genes arise from interspecies recombinations within a single gene and confer cephalosporin resistance through novel structural forms (15, 47), our findings of horizontally acquired, epistatically interacting structural and regulatory variants in *mtr* point to the potential complexity by which antibiotic resistance can arise through the interactions of multiple loci. Interspecies mosaicism will be an important consideration for future development of sequence-based molecular resistance diagnostics, as markers designed to amplify gonococcus-specific sequence will overlook or incorrectly diagnose resistance phenotype. Thus, as the number of commensal neisserial genome sequences increase, analyses that map the patterns and extent of interspecies recombination may be a valuable guide in understanding pathways to resistance and in designing appropriate diagnostic tools.

## MATERIALS AND METHODS

### Genome sequencing and population genomics.

Sequencing libraries were prepared using a modification of Illumina’s Nextera XP protocol (48). Samples were dual-indexed and pooled (*n* = 15 per pool). Paired-end 150-bp sequencing was conducted on an Illumina MiSeq (Illumina Corp., San Diego, CA) platform located at the Harvard T. H. Chan School of Public Health to an average depth of 40×. Previously sequenced read libraries were obtained from the NCBI’s Short Read Archive (project PRJEB2090) and the European Nucleotide Archive (projects PRJEB2999 and PRJEB7904) (8, 34).

To determine the impact of interspecific recombination at *mtrRCDE*, we assessed patterns of allelic diversity across the *mtrR* transcriptional repressor and the *mtrCDE* pump compared to the rest of the genome for the 1,102 GISP gonococcal isolates (8, 34). Reads were aligned to the FA1090 reference using Bowtie2 v.2.2.4 (49), and variants were called using pilon v.1.16 (50). Vcftools v.0.1.12 (51) was used to merge resultant vcf files and calculate genome-wide values of π and Tajima’s D over 100-bp sliding windows, and *r*^2^ linkage by site. Gubbins v.2.2.0 (52) was used to predict regions of elevated single-nucleotide polymorphism (SNP) densities. For each isolate, BLASTn was used to identify the top hit and highest percent sequence identity for *mtrD* and the *mtr* promoter to all *Neisseria* within the NCBI database (E value of <10^−40^).

The extent of exclusive ancestry between Neisseria gonorrhoeae, N. meningitidis, N. lactamica, and N. polysaccharea was assessed using the genealogical sorting index (*gsi*) (35) for each gene across a 25-kb window surrounding *mtrRCDE*. In brief, we downloaded *de novo* assemblies and raw sequencing reads from NCBI for N. meningitidis (*n* = 431), N. lactamica (*n* = 326), N. polysaccharea (*n* = 37), and N. gonorrhoeae (*n* = 1102) (8, 34). Raw reads were assembled with SPAdes v.3.7.0 (53), and assemblies were aligned to the N. gonorrhoeae FA1090 reference genome (AE004969.1) using progressiveMauve (54) (snapshot 2015-02-13 for linux-x64), since a multigenome alignment for all genomes was not computationally tractable. The sequences that were aligned to each gene within 25 kb of *mtrRCDE* in strain FA1090 were then extracted with custom Perl scripts (available on request) and realigned with MAFFT v.7.309 (55). This method of pairwise alignments of *de novo* assemblies to the FA1090 reference identifies orthologs between N. gonorrhoeae and the other *Neisseria* species using both sequence identity and microsynteny, which is conserved in the genomic region surrounding *mtrRCDE* (see Fig. S1 in the supplemental material). We then used RAxML v.8.1.4 (56) to reconstruct the phylogeny for each gene, using 50 bootstrap replicates and the GTRCAT substitution model. With these multispecies phylogenies, we calculated *gsi* with the *genealogicalSorting* R package (35, 57).

### Bacterial culture conditions.

N. gonorrhoeae isolates were provided by the CDC (Table 1). Isolates were cultured on GCB agar medium supplemented with 1% IsoVitaleX (Becton Dickinson Co., Franklin Lakes, NJ). After inoculation, the plates were incubated at 37°C in a 5% CO_2_ atmosphere incubator for 16 to 18 h. Antimicrobial susceptibility testing was conducted using the agar dilution method at a range of concentrations of azithromycin from 0 µg/ml to 16 µg/ml, crystal violet from 0.25 µg/ml to 32 µg/ml, Triton X-100 from 250 µg/ml to 16,000 µg/ml, and polymyxin B from 30 µg/ml to 1,000 µg/ml (1, 5). MICs were recorded after 24 h of growth. All isolate stocks were stored at −80°C in Trypticase soy broth containing 20% glycerol.

### Transformation of mosaic *mtr* alleles.

Genomic DNA was extracted from isolates by lysing growth from overnight plates in TE buffer (10 mM Tris [pH 8.0], 10 mM EDTA) with 0.5 mg/ml lysozyme and 3 mg/ml proteinase K (Sigma-Aldrich Corp., St. Louis, MO). DNA was purified using the PureLink Genomic DNA minikit, treated with RNase A (Thermo Fisher Corp., Waltham, MA), and stored in water. Primers were designed to amplify regions of *mtrRCDE*. For primer pairs that did not amplify over a region containing a DNA uptake sequence, to enhance transformation efficiency, the 12-bp AT-DNA uptake sequence (AT-DUS) was added to the forward primer (5′-ATGCCGTCTGAA-3′) (58). PCR was conducted in 50-µl volumes using Phusion High-Fidelity DNA polymerase (New England Biolabs, Ipswich, MA) using the conditions listed in Table S3 in the supplemental material. Amplified products were run on a 0.8% agarose gel, excised, and purified with the QIAEX II gel extraction kit (Qiagen Inc., Valencia, CA) to remove genomic DNA (gDNA) contamination.

Transformations were conducted in liquid culture as described previously (21, 36, 37) at 37°C in a 5% CO_2_ atmosphere incubator. In brief, we inoculated GCP broth (7.5 g protease peptone 3, 0.5 g soluble starch, 2 g dibasic K_2_HPO_4_, 0.5 g monobasic KH_2_PO_4_, 2.5 g NaCl, and double-distilled water [ddH_2_O] to 500 ml; Becton Dickinson Co., Franklin Lakes, NJ) supplemented with 1% IsoVitaleX and 10 µM MgSO_4_ (Sigma-Aldrich Corp., St. Louis, MO) with naturally competent piliated cells to an optical density (OD) of ~0.5. Suspensions were then incubated with approximately 1 µg of gDNA or purified PCR product for 10 min to allow DNA uptake and homologous recombination and subsequently spread on GCB with 1% IsoVitaleX and incubated for 4 h to allow for expression of novel alleles. Transformants resistant to azithromycin were selected by plating cells on GCB supplemented with 1% IsoVitaleX and 0.38 to 1 µg/ml azithromycin and picking single colonies after 18 h.

All transformants were screened for successful transformation by testing the levels of resistance to known substrates of the Mtr efflux pump system as well as sequencing to couple the acquisition of novel mosaic alleles with gain of resistance. Transformants generated with targeted PCR products from mosaic donors were screened for mosaic alleles by sequencing *mtrD* and the *mtr* promoter region via Sanger sequencing using the GeneWiz sequencing service (GeneWiz Inc., Cambridge, MA) (Table 2). Undirected gDNA-transformed cell lines were sequenced via Illumina whole-genome sequencing as described above. To detect the alleles gained from mosaic donors, we searched for SNP homology between the transformants and mosaic donors that was not present in the 28Bl recipient strain (Fig. 3). Genomes were *de novo* assembled using SPAdes v.3.7.0 (53) and annotated with prokka v.1.11 (59). Alignment of transformant libraries to the 28Bl and mosaic references was conducted in Bowtie2 v.2.2.4 (49) using the “end-to-end” and “very-sensitive” options, which resulted in a higher percentage of uniquely mapped reads than any other combination of preset options. SNPs and indels between read libraries and the reference genomes were called with pilon v.1.16 (50), removing variant calls with qualities of <15.

### Transcriptome construction.

Cells harvested from overnight plates were suspended in GCP supplemented with 1% IsoVitaleX and 0.042% sodium bicarbonate. Cultures were incubated at 37°C for 2 h to mid-log phase and then exposed to a sublethal dose of azithromycin (AZI) (0.125 µg/ml). RNA was extracted at 0 min (pre-AZI) and 120 min (post-AZI) exposure using the Direct-Zol kit (Zymo Research, Irvine, CA). Transcriptome libraries were prepared at the Broad Institute at the Microbial ‘Omics Core using a modified version of the RNAtag-seq protocol (60). Five hundred nanograms of total RNA was fragmented, depleted of genomic DNA, dephosphorylated, and ligated to DNA adapters carrying 5′-AN_8_-3′ barcodes of known sequence with a 5′ phosphate and a 3′ blocking group. Barcoded RNAs were pooled and depleted of rRNA using the RiboZero rRNA depletion kit (Epicentre, Madison, WI). Pools of barcoded RNAs were converted to Illumina cDNA libraries in two main steps: (i) reverse transcription of the RNA using a primer designed to the constant region of the barcoded adapter with addition of an adapter to the 3′ end of the cDNA by template switching using SMARTScribe (Clontech, Mountain View, CA) as described previously (61); (ii) PCR amplification using primers whose 5′ ends target the constant regions of the 3′ or 5′ adapter and whose 3′ ends contain the full Illumina P5 or P7 sequences. cDNA libraries were sequenced on the Illumina Nextseq 500 platform to generate 50-bp paired-end reads.

Barcode sequences were removed, and reads were aligned to the FA1090 reference genome. Read counts were assigned to genes and other genomic features using custom scripts. For the FA1090 reference genome, we mapped reads to either the sense or antisense strand for coding domain sequences (CDSs) (*n* = 1,894), tRNAs (*n* = 55), and rRNAs (*n* = 12). For intergenic regions (IGRs) (*n* = 1,722), we mapped to each antiparallel strand. Differential expression analysis was conducted in DESeq2 v.1.10.1 (62).

### Data availability.

All genomic and transcriptomic read libraries generated in this study are available from the NCBI SRA database (accession numbers PRJNA475134 and PRJNA475139); all Sanger sequencing files are available in the NCBI database (accession numbers MH576918 to MH576947).
